# Double tibial tunnels ‘supra’ over‐the‐top anterior cruciate ligament reconstruction with lower initial graft tension achieves comparable anterior stability to single‐tunnel: A cadaveric study using a robotic simulator

**DOI:** 10.1002/jeo2.70466

**Published:** 2025-12-01

**Authors:** Kousuke Shiwaku, Tomoyuki Suzuki, Konsei Shino, Satoshi Yamakawa, Hidenori Otsubo, Daisuke Suzuki, Shinichiro Okimura, Katsunori Takahashi, Shogo Nabeki, Hirofumi Ohnishi, Hiromichi Fujie, Atsushi Teramoto

**Affiliations:** ^1^ Department of Orthopedic Surgery Sapporo Medical University School of Medicine Sapporo Japan; ^2^ Sapporo Maruyama Orthopaedic Hospital Sapporo Japan; ^3^ Sports Orthopaedic Center Yukioka Hospital Osaka Japan; ^4^ Department of Sports Medical Biomechanics, Graduate School of Medicine Osaka University Osaka Japan; ^5^ Sapporo Sports Clinic Sapporo Japan; ^6^ Department of Health Science Hokkaido Chitose Collage of Rehabilitation Chitose Japan; ^7^ Department of Public Health Sapporo Medical University School of Medicine Sapporo Japan; ^8^ Department of Mechanical Engineering, Graduate School of Science and Engineering Tokyo Metropolitan University Tokyo Japan

**Keywords:** ACL reconstruction, biomechanics, cadaveric knee, double bundle (DB), open physis, robotic system, ‘supra’‐over‐the‐top (s‐OTT)

## Abstract

**Purpose:**

To compare the anterior tibial translation (ATT) between single‐bundle (SB) and double‐bundle (DB) ‘supra’‐over‐the‐top anterior cruciate ligament reconstruction (s‐OTT ACLR) during the anterior drawer test in a cadaveric model.

**Methods:**

This was a controlled laboratory study. Seven fresh‐frozen cadaveric knee specimens and a robotic testing system were used. Initially, the same testing procedure (100 N anterior drawer load applied at 0°, 15°, 30°, 60° and 90° of knee flexion) was applied to the following four different knee conditions: intact, anterior cruciate ligament (ACL)‐sectioned, SB s‐OTT ACLR and DB s‐OTT ACLR knees. Subsequently, a combined load of 5 Nm internal tibial torque and 10 Nm valgus torque was applied at 15° and 30° of knee flexion as a simulated pivot shift. During the tests, anterior tibial translation (ATT) was recorded. The initial tension was set at 22 N for the SB s‐OTT ACLR and 11 N for each graft in the DB s‐OTT ACLR at 30° of knee flexion. For the SB s‐OTT ACLR with an 8‐mm‐diameter tibial tunnel, the drilling area was 16 Π mm² (4 × 4 × Π). Regarding the DB s‐OTT ACLR with two 5‐mm‐diameter tibial tunnels, the total drilling area was 12.5 Π mm² (2.5 × 2.5 × Π × 2).

**Results:**

Both SB and DB s‐OTT ACL reconstructions significantly reduced ATT compared with the ACL‐sectioned state under both anterior drawer and simulated pivot‐shift loads, while showing no difference from the intact knee. No significant ATT difference was detected between SB and DB s‐OTT ACLRs at any tested flexion angle, and DB achieved anterior knee stability comparable to SB despite its lower initial graft tension.

**Conclusions:**

DB s‐OTT ACLR demonstrated ATT and in situ graft force comparable to SB s‐OTT ACLR in a cadaveric robotic study. These findings suggest that DB s‐OTT ACLR can achieve anterior knee stability similar to SB s‐OTT ACLR with lower initial graft tension.

**Level of Evidence:**

N/A.

**Abbreviations**
ACL: anterior cruciate ligament
ACLR: anterior cruciate ligament reconstruction
ATT: anterior tibial translation
DB: double‐bundle
PL: posterolateral
SB: single‐bundle
s‐OTT: supra‐over‐the‐top

## INTRODUCTION

Anterior cruciate ligament (ACL) injury is common in sports and is usually treated surgically [26, 34, 36]. Although anatomical ACL reconstruction (ACLR) commonly yields good clinical outcomes, including good anterior and rotatory knee stability and a higher likelihood of return to sports activities [26], this procedure may pose risks for skeletally immature patients with open physes [5, 8] or may be unsuitable for revision ACLR with large widened primary ACLR tunnels [41].

The number of ACLRs in skeletally immature patients has been increasing to prevent secondary meniscal or chondral injuries following unsuccessful nonoperative treatment [19, 21, 28]. A major concern for ACLR in skeletally immature patients is physeal damage due to tunnel drilling, which may result in angular deformity or leg length discrepancy [5, 8, 30]. These complications are more frequently observed in the femur than in the tibia [7] because the femoral physis closes later than the tibial physis, and the femoral tunnel's orientation is less perpendicular to the physis than that of the tibial tunnel [5].

In patients undergoing revision ACLR, enlarged tunnels around the ACL attachment sites may cause difficulty in properly positioning the grafts within the sites. Thus, residual anterior or rotatory knee instability is frequently observed in patients with markedly widened tunnels after one‐stage revision ACLR with improperly‐placed grafts or extended treatment durations are required for those undergoing two‐stage revision ACLR [41]. This problem occurs more frequently in the femoral tunnel because tunnel widening is more prominent in the femur than in the tibia [16].

For the above‐mentioned cases, considerable attention has been drawn to over‐the‐top (OTT) ACLR as it does not require femoral tunnel creation [14]. Instead, a graft is secured at the lateral femoral metaphysis proximal to the posterior femoral condyle, known as the OTT position [1, 9, 20, 24, 27]. Although satisfactory clinical outcomes [9, 25, 27, 31, 40] and good anterior stability at lower knee flexion angles in biomechanical testing using cadavers [17] have been reported, increased anterior laxity has been observed at deeper flexion angles in both biomechanical testing and clinical physical examinations [24, 25]. Thus, Shiwaku et al. modified the surgical procedure to shift the femoral fixation site proximally to prevent graft slippage at deeper knee flexion and named it ‘supra’‐OTT (s‐OTT) ACLR [35]. Shiwaku et al. previously reported that the single‐bundle (SB) s‐OTT ACLR procedure results in less anterior laxity than the conventional OTT procedure at higher flexion angles [35].

While a previous study on s‐OTT ACLR used a single tibial tunnel (SB s‐OTT ACLR), the procedure with double tibial tunnels (double‐bundle [DB] s‐OTT ACLR) may have potential advantages over SB s‐OTT ACLR, as indicated by previous studies [3, 11, 13, 15, 32, 33]. The primary aim was to compare of anterior tibial translation (ATT) between DB and SB s‐OTT ACLR during the anterior drawer test. The secondary aim was to compare of anterior stability between DB and SB s‐OTT ACLR during the pivot shift test. The corresponding hypotheses were as follows: (1) DB s‐OTT ACLR would demonstrate comparable ATT compared to SB s‐OTT ACLR in the anterior drawer test although initial tension was lower in DB s‐OTT ACLR; (2) DB s‐OTT ACLR would demonstrate comparable anterior and rotatory knee stability compared to SB s‐OTT ACLR in the pivot shift test although initial tension was lower in DB s‐OTT ACLR.

## METHODS

### Testing apparatus

**Figure 1:**
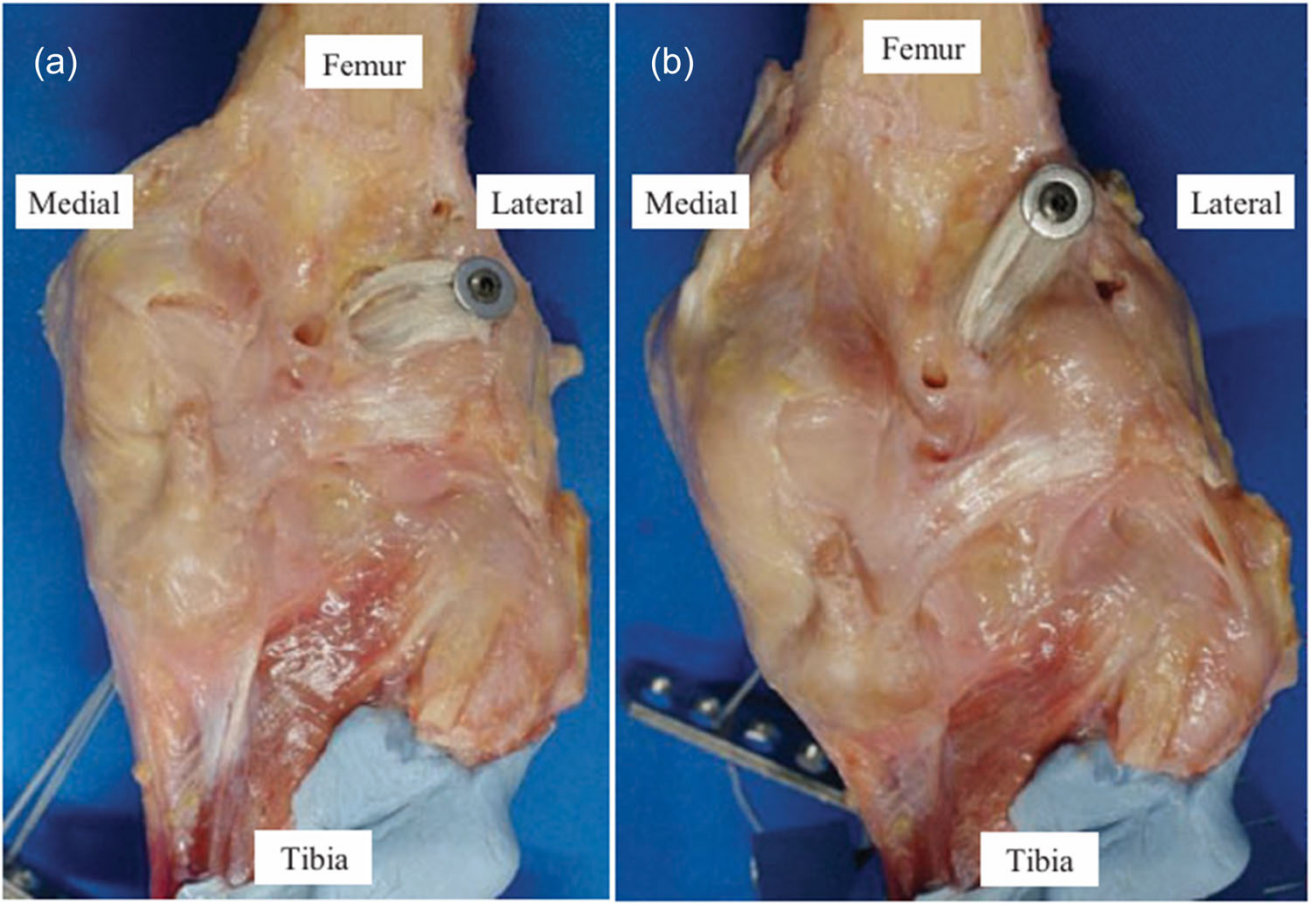
Anterior cruciate ligament reconstruction of the right knee (posterior view). (a) Conventional over‐the‐top (OTT) and (b) supra‐OTT. Reprinted from Shiwaku et al. with permission. Published under the Creative Commons CC BY‐NC‐ND 4.0 license. The Orthopaedic Journal of Sports Medicine. Figure reproduced without modification.

A robotic testing system was utilised with a custom‐made manipulator with six degrees of freedom, equipped with a universal force–torque sensor (DELTA IP65; SI‐660‐60; ATI Industrial Automation; Figure 1) [38, 39]. This robotic system was used to simulate physiological knee joint motion in vitro using the joint coordinate system developed by Grood and Suntay, with axes corresponding to flexion‐extension, internal‐external rotation and varus‐valgus motion [10]. This system, which guides the displacement of and force/torque applied to the knee joints, was controlled in real‐time using a LabView (v12.0.1; National Instruments)‐based program.

### Specimen preparation

The study protocol obtained institutional review board approval for the acquisition, use, and disposal of fresh‐frozen human cadaveric knees (approval number: 1‐2‐68). Seven fresh‐frozen knee specimens from six male and one female cadavers were utilised (mean age at death, 83.9 years; range, 70–90 years). All specimens were obtained from donors who had provided informed consent prior to death. Physical examinations were conducted before biomechanical testing to confirm ligamentous stability and range of motion from full extension to 130° of flexion. Knees exhibiting an extension deficit >5° or a maximum flexion <130° were excluded. This threshold follows previous cadaveric biomechanical studies that require reproducible full‐extension kinematics and deep‐flexion analysis up to 130° [2, 12, 18, 23]. Range of motion was verified with a goniometer prior to testing. Specimens with ligamentous instability, meniscus injury, or history of knee surgery were excluded. Cartilage and meniscal degeneration were assessed using the Outerbridge and Stoller classifications [29, 37], respectively, and all included knees demonstrated grade 1 or less in both systems.

Specimens were thawed at room temperature for a minimum of 24 h prior to testing. All biomechanical tests were completed within approximately 10 h from initiation to completion. To preserve the integrity of soft tissues, all specimens were tested under standardised environmental conditions. Subsequently, the specimens were continuously moistened to prevent tissue desiccation. The femur and tibia were cut to at least 15 cm above and below the joint line, whereas the fibula was cut 5 cm below the proximal tibiofibular joint. All muscles (except the popliteus) and the patella were removed. The joint capsule, ligaments and menisci were preserved. Both ends of the tibia and femur were fixed using acrylic resin (Ostron II; GC) poured into a cylindrical mold. The fibula was fixed in its original position using resin. The femoral and tibial cylinders were fixed using aluminium clamps and mounted onto the robotic system's end‐effector [6] (FRS‐2010; Technology Service; Figure 1).

The manipulator, which operated with the universal force–torque sensor, was placed on the end‐effector. The tibia was fixed on the end‐effector, and the femur was anchored to the base of the apparatus using metal clamps. An adjustable tension rod was attached to the plate, which was connected to the tibial clamp, and the force gauge was connected to the free ends of the graft.

### Surgical procedure

Both SB [37] and DB s‐OTT ACLRs were applied to the same knee. First, SB s‐OTT ACLR was performed for each knee, followed by DB s‐OTT ACLR after the previous tunnel was snugly filled with autogenous cancellous bone. This sequence of reconstructions was standardised to minimise the effect of repeated tibial tunnel drilling.

The same hamstring graft was used in both procedures. The graft was made from doubled semitendinosus and gracilis tendons. This graft used four strands in the SB s‐OTT ACLR and two strands per tendon in the DB s‐OTT ACLR. The free ends of the grafts were sutured using No. 3 polyester sutures with the Krackow suture technique.

**Figure 2:**
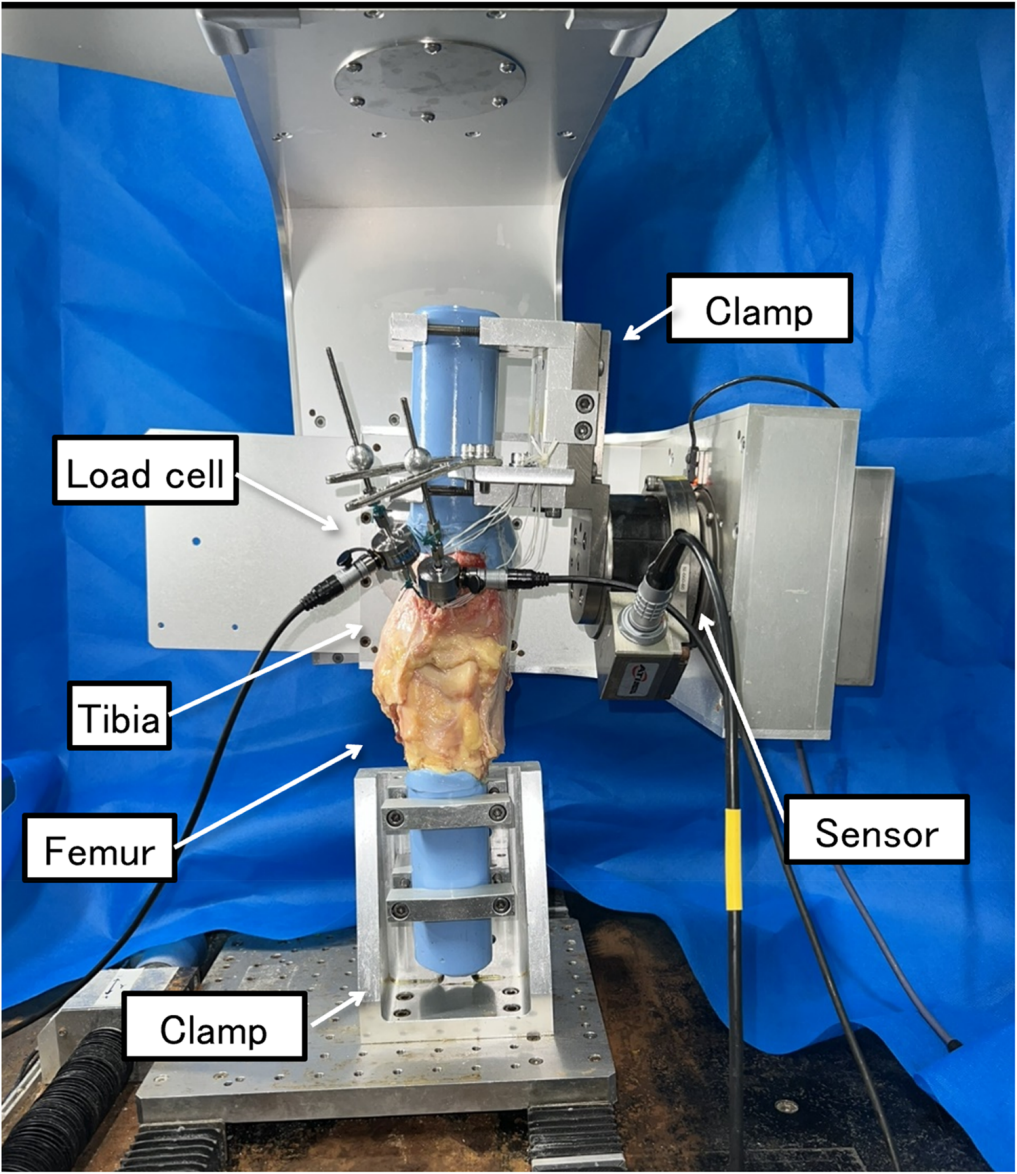
Robotic testing system shown with a right knee. The manipulator, which works with the universal force‐torque sensor, is placed on the end‐effector. The tibia is fixed on the end‐effector, and the femur is fixed to the lower part of the device using metal clamps. An adjustable tension rod is attached to the plate, which is connected to the tibial clamp, and the force gauge is attached to the tendon graft.

After fully removing the native ACL, the graft fixation to the femur for supra‐OTT ACLR was performed at approximately 2 cm proximal to the conventional OTT position, which was around the proximal insertion of the lateral head of the gastrocnemius and the lateral edge of the posterior cortex (Figure 2) [35]. After identifying the fixation points, a guidewire was drilled into each point (Smith & Nephew). The posterior joint capsule was penetrated around the OTT positions by passing a curved clamp in an inside‐out manner. The capsule was incised longitudinally and proximally extended until the femoral cortex was reached, providing direct visualisation of both femoral insertion sites. To avoid altering graft trajectory during flexion–extension, the capsule was intentionally left open throughout all testing conditions. AS shown in Figure 2, graft passage and fixation were performed under full visual control. The graft's looped end was passed through. The looped portion of the hamstring graft was secured using a cannulated screw with a washer (Smith & Nephew).

For the SB s‐OTT ACLR, to create one tibial tunnel, a guidewire was inserted into the centre of the ACL attachment area referencing the anterior intercondylar ridge anteriorly, the medial intercondylar ridge medially, and the anterior horn of the lateral meniscus laterally from the anterior aspect of the tibia using a tibial guide; the guidewire was over‐drilled to the size of the tendon graft (8 mm). After the tests for SB s‐OTT ACLR were performed, the tunnel was snugly filled with autogenous cancellous bone.

Autogenous cancellous bone was harvested as a cylindrical plug from the anteromedial tibial tuberosity. The plug was fashioned 1 mm larger in diameter than the existing tunnel and trimmed to the tunnel length before being press‐fitted into the defect. An anterior–posterior load of approximately 120 N was manually applied to confirm the absence of displacement and secure initial fixation. This approach has been reported to restore adequate stability for biomechanical testing [34, 38, 39].

**Figure 3:**
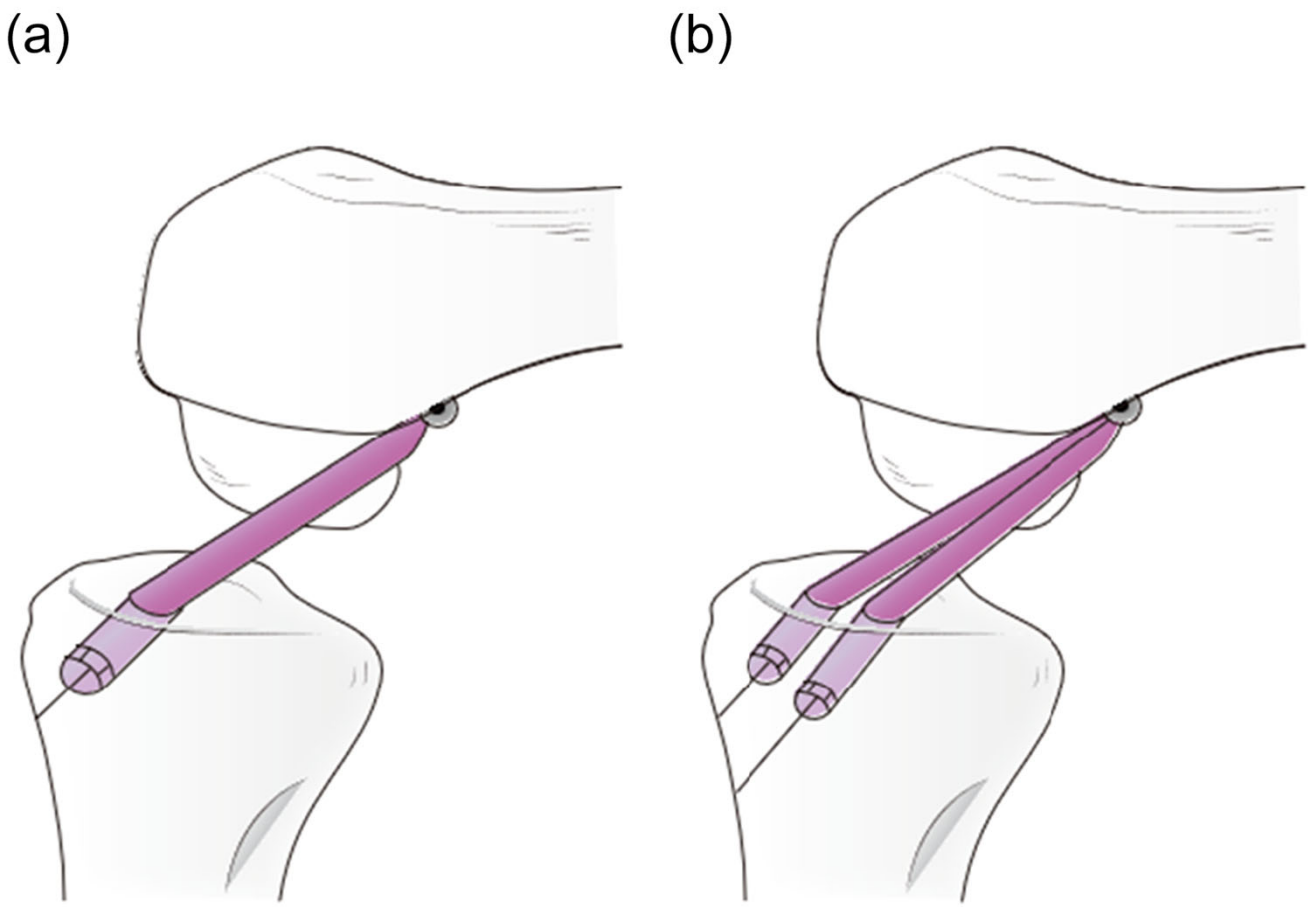
Schema of SB and DB s‐OTT ACLR of the right knee. Medial view without medial femoral condyle. (a) SB s‐OTT ACLR and (b) DB s‐OTT ACLR. ACLR, anterior cruciate ligament reconstruction; DB s‐OTT, double‐bundle supra‐over‐the‐top; SB s‐OTT, single‐bundle supra‐over‐the‐top.

For the DB s‐OTT ACLR (Figure 3), to create two tibial tunnels, two guidewires were inserted into the centre of the anteromedial and posterolateral (PL) bundle‐attachment area along the medial intercondylar ridge and were over‐drilled to the size of the tendon graft (5 mm). To monitor the graft's tension in real time, the sutures of the free ends of the graft were connected to the force gauge installed at the tibial tunnel entrance (Figure 1).

Regarding tunnel area, for SB s‐OTT ACLR with an 8 mm diameter tibial tunnel, the area of drilling was 16 Π mm² (4 × 4 × Π). Regarding DB s‐OTT ACLR with two 5 mm diameter tibial tunnels, the total area of drilling was 12.5 Π mm² (2.5 × 2.5 × Π × 2).

### Testing protocol

For graft preconditioning, the specimen was set at 30° of knee flexion on the robot, and a 50‐N tensile preload was applied to the graft for 300 s to minimise viscoelastic creep effects. The initial tension of the grafts was determined based on findings from two previous studies with similar biomechanical testing setups. One of the previous studies on SB s‐OTT ACLR [35] demonstrated that applying an initial tension of 44 N for the graft led to over‐constraining. The other study on ACLR with femoral and tibial tunnels [39] investigated the laxity‐match pretension, which is the amount of graft tension required for the reconstructed knee to display the same amount of anterior laxity as the intact knee at 30° of knee flexion. The study demonstrated that the laxity match pretension for SB ACLR with one femoral and one tibial tunnel was 26.3 N, while that for DB ACLR with two femoral and two tibial tunnels was 5.6 N [39].

Considering these results, 22 N at 30° of knee flexion of the initial tension for the SB s‐OTT ACLR was selected in our study. Accordingly, each initial tension for the two grafts of the DB s‐OTT ACLR, which involves two tibial tunnels, was adjusted as 11 N at 30° of knee flexion to ensure that the total tension of the grafts matched that of the SB s‐OTT ACLR. After the tests of the first SB s‐OTT ACLR, the graft was removed. Subsequently, the same preparations and tests were repeated for the DB s‐OTT ACLR.

First, the flexion–extension axis of the knee was defined as 0° of flexion when 0.5 Nm of extension moment was applied to the intact knee. Next, passive flexion–extension was carried out from its hyperextended position, under a 5 Nm extension moment, to 120° of knee flexion; this was applied at a rate of 0.5°/s and repeated for three cycles for preconditioning. Finally, two types of external loading were applied: first, 100 N of anterior tibial load was applied to the knee at 0°, 15°, 30°, 60° and 90° of flexion to simulate the Lachman test or the anterior drawer test; second, a combined load of 5 Nm of internal tibial torque and 10 Nm of valgus torque was applied to the knee at 15° and 30° of flexion, respectively, as a simulated pivot shift test [38, 39]. Both tests were performed under position‐based control to ensure a consistent reproduction of joint kinematics. Following testing of the intact knee, the ACL was resected. Subsequently, both loading tests were conducted, and the recorded intact knee motion was reproduced in the ACL‐resected knee, while the force/torque of the knee was recorded. The in situ force in the ACL was calculated based on the principle of superposition [4, 5] using the six‐degree‐of‐freedom force/torque data of the intact and resected‐ACL states. Next, the SB s‐OTT ACLR was performed. An anterior drawer load test and a simulated pivot shift test were performed in each OTT ACLR condition. The three‐dimensional motion and the in situ force for each graft, measured using the force gauge, were recorded [35, 38, 39]. Finally, the DB s‐OTT ACLR was performed in the same knee, and the same tests were performed.

### Statistical analysis

The ATT under both loads in the intact, ACL‐resected, SB s‐OTT ACLR and DB s‐OTT ACLR conditions were analysed using a two‐factor repeated‐measures analysis of variance followed by Bonferroni‐adjusted pairwise comparisons to assess the effects of knee condition and flexion angle. The in situ forces of the ACL and SB s‐OTT ACLR graft were also analysed using a two‐factor repeated‐measures analysis of variance with post hoc pairwise comparison.

All statistical analyses were performed using IBM SPSS Statistics for Windows, version 28.0 (IBM Corp.). For ATT and in situ force, all *p*‐values for significant differences were defined as <0.008 with Bonferroni correction. Post hoc power analysis was performed to determine the power of the study. Based on the mean values and standard deviations of the ATT and in situ force during the anterior drawer load, the power of this study was 0.807–1.000.

## RESULTS

### ATT

**Figure 4:**
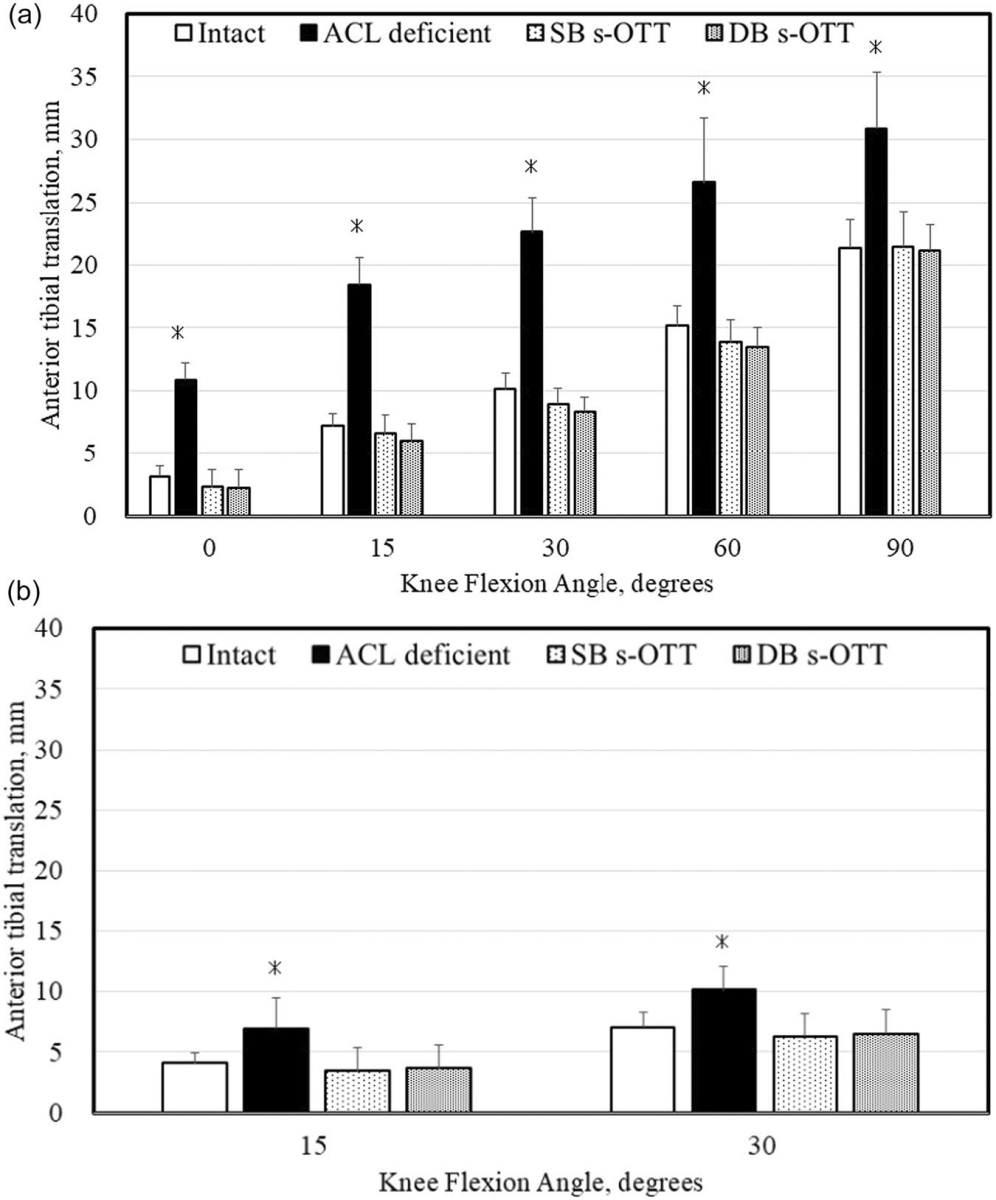
Anterior tibial translation at each knee flexion angle tested in each knee state. (a) Anterior tibial translation tested under 100 N of anterior tibial load and (b) under simulated pivot shift test (internal rotation of 5 Nm plus valgus of 10 Nm). * Significantly different from all other states. ^#^ Significantly different between two groups. ACL, anterior cruciate ligament; DB s‐OTT, double‐bundle supra‐over‐the‐top; SB s‐OTT, single‐bundle supra‐over‐the‐top.

The ATT under each load is shown in Figure 4 and Table 1. In the intact state, ATT increased progressively with greater knee flexion angles and showed a marked increase following ACL resection under anterior tibial load or the simulated pivot shift test at each flexion angle (*p* < 0.008). ATT values in knees reconstructed with SB and DB s‐OTT ACLR procedures under anterior tibial load or simulated pivot shift test at each flexion angle were significantly smaller than those with the ACL‐sectioned state (*p* < 0.003) and did not differ significantly from the intact condition (n.s.).

**Table 1:** Anterior tibial translation at each knee flexion angle.

	Intact		ACL deficient		SB s‐OTT		DB s‐OTT	
	Mean	SD	Mean	SD	Mean	SD	Mean	SD
Anterior tibial load								
0°	3.2	0.9	10.9	1.4	2.3	1.4	2.3	1.9
15°	7.2	1.0	18.4	2.1	6.6	1.6	6.0	2.8
30°	10.1	1.2	22.6	2.8	9.0	1.7	8.4	2.8
60°	16.2	1.6	26.6	5.1	13.9	2.0	13.8	2.6
90°	24.0	2.3	30.9	4.4	21.4	2.6	21.1	2.7
Simulated pivot shift								
15°	4.1	0.9	7.0	2.5	3.4	1.9	3.7	1.9
30°	7.1	1.2	10.2	1.9	6.3	2.0	6.5	1.8

*Note*: Anterior tibial translation at each knee flexion angle was tested in each knee state: (a) under 100 N of anterior tibial load and (b) under simulated pivot shift test (internal rotation of 5 Nm plus valgus of 10 Nm).

Abbreviations: ACL, anterior cruciate ligament; DB s‐OTT, double‐bundle supra‐over‐the‐top; SB s‐OTT, single‐bundle supra‐over‐the‐top.

### In situ force

**Figure 5:**
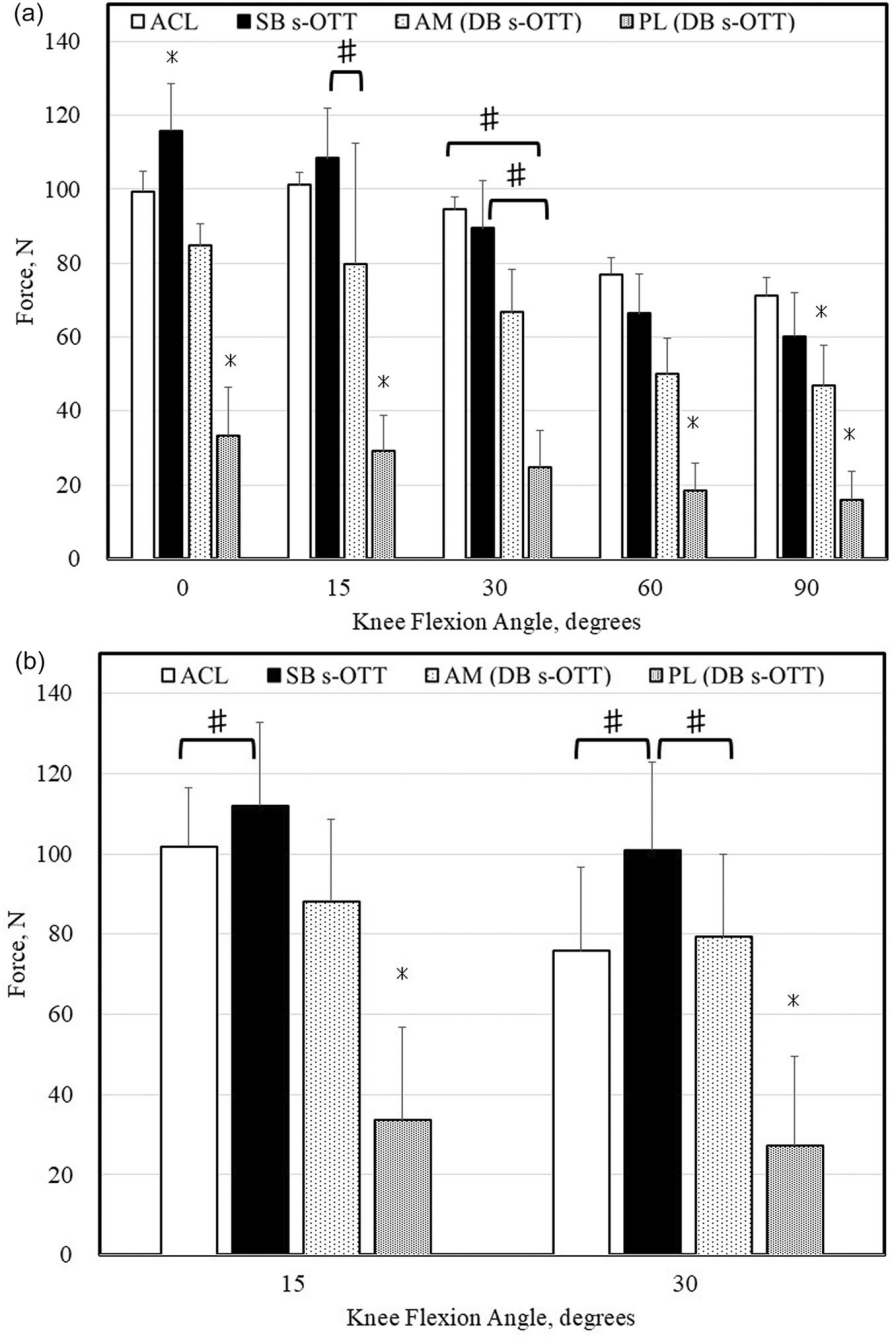
In situ forces of the ACL and OTT grafts at each knee flexion angle. (a) Forces of the ACL and OTT grafts tested under 100 N of anterior tibial load and (b) under simulated pivot shift test (internal rotation of 5 Nm plus valgus of 10 Nm). * Significantly different from all others. ^#^ Significantly different between two groups. ACL, anterior cruciate ligament; DB s‐OTT, double‐bundle supra‐over‐the‐top; SB s‐OTT, single‐bundle supra‐over‐the‐top; AM, anteromedial; PL, posterolateral.

The in situ forces under each loading condition are shown in Figure 5 and Table 2. Under anterior tibial load, the in situ force of the native ACL remained relatively constant between 0° and 30° of knee flexion but progressively decreased at higher knee flexion angles. In the SB s‐OTT ACLR group, the graft in situ force at 0° of flexion was significantly greater than that of the native ACL, AM bundle and PL bundle (*p* < 0.008). At 15°, this force was also significantly greater than that of the AM and PL bundles (*p* < 0.007). In the DB s‐OTT ACLR group, the in situ force of the PL bundle was significantly lower than that of the native ACL, SB s‐OTT graft, and AM bundle at all tested flexion angles under anterior tibial load (*p* < 0.008), except at 30° when compared to the AM bundle (n.s.). During the simulated pivot‐shift test, the in situ force of the PL bundle in the DB s‐OTT ACLR group was significantly lower than that of the native ACL, SB s‐OTT graft and AM bundle at all tested flexion angles (*p* < 0.005).

**Table 2:** In situ forces of the ACL and OTT grafts.

	ACL		SB s‐OTT		DB s‐OTT					
					AM		PL		Total	
	Mean	SD	Mean	SD	Mean	SD	Mean	SD	Mean	SD
Anterior tibial load										
0°	99.4	5.5	115.9	12.6	84.7	24	33.3	17.6	118	25.2
15°	101.1	3.5	94.1	38.8	79.8	21.9	29.2	15.7	109	17.7
30°	94.4	3.5	89.5	12.8	66.7	20	24.7	12.6	91.4	15.3
60°	76.9	4.6	66.5	10.6	50	14.3	18.6	6.6	68.5	10.5
90°	71.2	4.8	60.2	11.9	46.9	12.9	16.1	6.9	63	10.5
Simulated pivot shift										
15°	101.8	14.7	112	20.8	88.2	27.5	33.6	15.2	121.8	30.7
30°	75.8	20.8	101	21.9	79.3	22.1	27.4	12.7	106.6	26.7

*Note*: In situ forces of the ACL and OTT grafts at each knee flexion angle were tested in each knee state: (a) under 100 N of anterior tibial load and (b) under simulated pivot shift test (internal rotation of 5 Nm plus valgus of 10 Nm).

Abbreviations: ACL, anterior cruciate ligament; AM, anteromedial; DB s‐OTT, double‐bundle supra‐over‐the‐top; PL, posterolateral; SB s‐OTT, single‐bundle supra‐over‐the‐top.

### Other data

The other data are shown in Figures S1 and S2.

## DISCUSSION

The most important finding was that DB s‐OTT ACLR with two smaller diameter tibial tunnels showed comparable anterior and rotatory knee stability to SB s‐OTT ACLR with a single larger diameter tunnel. From a biomechanical perspective, as illustrated in Figure 3, there is a disadvantage for DB s‐OTT because the running route of the PL graft from the femoral OTT fixation point to the tibial PL tunnel is more vertical than that of the PL part of the SB s‐OTT graft. Despite this disadvantage, DB s‐OTT ACLR with lower initial tension for each graft and smaller diameter tibial tunnels showed equal anterior‐posterior knee laxity compared with SB s‐OTT ACLR. This discrepancy could be attributed to the fact that the graft deformation inside the tibial tunnel in the DB procedure is smaller than that in the SB procedure [32, 33].

Regarding initial tension, some studies have reported that SB conventional OTT ACLR with an initial tension of 44 N at 30° of knee flexion [20] tends to over‐constrain the knee. This is similar to the SB s‐OTT ACLR with an initial tension of 44 N at 30° of knee flexion in the previous study [35]. Therefore, the 22 N at 30° of knee flexion of initial tension of SB s‐OTT ACLR was selected for this study. Considering the result that the ATT of SB s‐OTT ACLR was equivalent to that of the intact knee, the SB s‐OTT ACLR knees in this study were not over‐constrained, suggesting that the initial tension of 22 N was adequate. Similarly, since the initial tension of 11 N at 30° of knee flexion was applied to each bundle of the DB s‐OTT graft, the DB s‐OTT ACLR knees were also not over‐constrained.

For skeletally immature patients, DB s‐OTT ACLR has an additional benefit in reducing the risk of physeal damage. The risk of physeal damage is positively correlated with the area of drilling [22]. This theoretical advantage may have particular clinical significance.

The use of a supra‐physeal femoral fixation and two smaller tibial tunnels in DB s‐OTT ACLR may reduce the risk of physeal injury compared to traditional transphyseal ACLR. This advantage is particularly important for skeletally immature patients with open physes, where preserving growth potential is critical.

Thus, the DB s‐OTT ACLR technique not only maintains anterior and rotatory knee stability comparable to SB s‐OTT ACLR but also offers a safer reconstructive option for paediatric and adolescent populations.

Regarding initial tension, some studies have reported that SB conventional OTT ACLR with an initial tension of 44 N at 30° of knee flexion [20] tends to over‐constrain the knee. This is similar to the SB s‐OTT ACLR with an initial tension of 44 N at 30° of knee flexion in the previous study [35]. Therefore, the 22 N at 30° of knee flexion of initial tension of SB s‐OTT ACLR was selected for this study. Considering the result that the ATT of SB s‐OTT ACLR was equivalent to that of the intact knee, the SB s‐OTT ACLR knees in this study were not over‐constrained, suggesting that the initial tension of 22 N was adequate. Similarly, since the initial tension of 11 N at 30° of knee flexion was applied to each bundle of the DB s‐OTT graft, the DB s‐OTT ACLR knees were also not over‐constrained.

Another study on ACLR with femoral and tibial tunnels using a similar biomechanical testing setup investigated the laxity match pretension, which is the amount of graft tension required for the reconstructed knee to display the same amount of anterior laxity as the intact knee at 30° of flexion [39]. The study demonstrated that the laxity match pretension for SB ACLR with one femoral and one tibial tunnel was 26.3 N, while that for DB ACLR with two femoral and two tibial tunnels was 5.6 N. Thus, the total initial tension of 22 N applied in our study was considered appropriate.

### Limitations

This study had some limitations. First, anatomical ACLR via tunnels was not performed for comparison; however, previous comparative studies showed little to no difference between anatomical and OTT ACLRs [1, 17, 20, 24]. Second, although the order of the surgical procedures was not randomised, close attention was paid to fitting the bone graft snugly into the tunnel and confirming that no tendon deterioration occurred. If the tunnel or tendon graft deteriorated even slightly, the specimen was excluded from analysis. Third, no tunnel breakage or graft damage was observed macroscopically during or after the experiments. The experiments were performed using specimens from older donors with poor bone quality and tendon degeneration, which could have caused potential biases. Fourth, this experimental model used the same knee joint and tendons multiple times. However, using the same hamstring grafts in the same knees in two different types of OTT ACLR enabled the two procedures to be compared with minimised interspecimen variation and increased statistical power. Fifth, although DB s‐OTT ACLR restored anterior‐posterior knee stability within the physiological range, potential limitations in rotatory stability or motion in other directions cannot be completely ruled out. Finally, as these results were obtained through in vitro tests, muscle forces, graft remodelling, load relaxation and the contribution of the soft tissues, including the anterior capsule and extensor mechanism, which had been excised during specimen preparation, were not considered. Moreover, the simulated pivot shift, comprising coupled moments at a static flexion angle, may not have sufficiently replicated the in vivo kinematics of the pivot shift test, even though such biomechanical tests have been used in numerous studies [38, 39].

## CONCLUSIONS

DB s‐OTT ACLR demonstrated comparable ATT and in situ graft force to SB s‐OTT ACLR in a cadaveric robotic study. These findings suggest that DB s‐OTT ACLR can achieve anterior knee stability similar to SB s‐OTT ACLR with lower initial graft tension.

## AUTHOR CONTRIBUTIONS

Konsei Shino wrote the initial draft of the manuscript. Kousuke Shiwaku, Tomoyuki Suzuki, Hidenori Otsubo, Satoshi Yamakawa, Konsei Shino, Hidenori Otsubo, Hiromichi Fujie and Atsushi Teramoto designed the study, contributed to data analysis and interpretation, and assisted in the preparation of the manuscript. Konsei Shino, Katsunori Takahashi, Shogo Nabeki, Shinichiro Okimura and Daisuke Suzuki have contributed to data collection. All other authors have contributed to interpretation, and critically reviewed the manuscript. All authors approved the final version of the manuscript and agree to be accountable for all aspects of the work in ensuring that questions related to the accuracy or integrity of any part of the work are appropriately investigated and resolved.

## CONFLICT OF INTEREST STATEMENT

The authors declare no conflicts of interest.

## ETHICS STATEMENT

The authors have nothing to report.

## Supporting information


**Supplemental Figure 2** Relative position of tibia in each knee status under anterior tibial load. (a) Internal rotation, (b) valgus rotation, (c) lateral shift, and (d) proximal displacement. The error bars indicate 1 SD of the sample mean. ACLR, anterior cruciate ligament; DB s‐OTT, double‐bundle supra‐over‐the‐top; SB s‐OTT, single‐bundle supra‐over‐the‐top.

**Supplemental Figure 1** Relative position of tibia in each knee status under anterior tibial load. (a) Internal rotation, (b) valgus rotation, (c) lateral shift, and (d) proximal displacement. The error bars indicate 1 SD of the sample mean. ACLR, anterior cruciate ligament; DB s‐OTT, double‐bundle supra‐over‐the‐top; SB s‐OTT, single‐bundle supra‐over‐the‐top.

## Data Availability

The authors have nothing to report.
