# Supplementary figures and images for: Double tibial tunnels ‘supra’ over‐the‐top anterior cruciate ligament reconstruction with lower initial graft tension achieves comparable anterior stability to single‐tunnel: A cadaveric study using a robotic simulator
Source: J Exp Orthop. 2025 Dec 1;12(4):e70466. doi: 10.1002/jeo2.70466 (PMC12666155; doi:10.1002/jeo2.70466)

**Supplenmental Fig. 2**

a


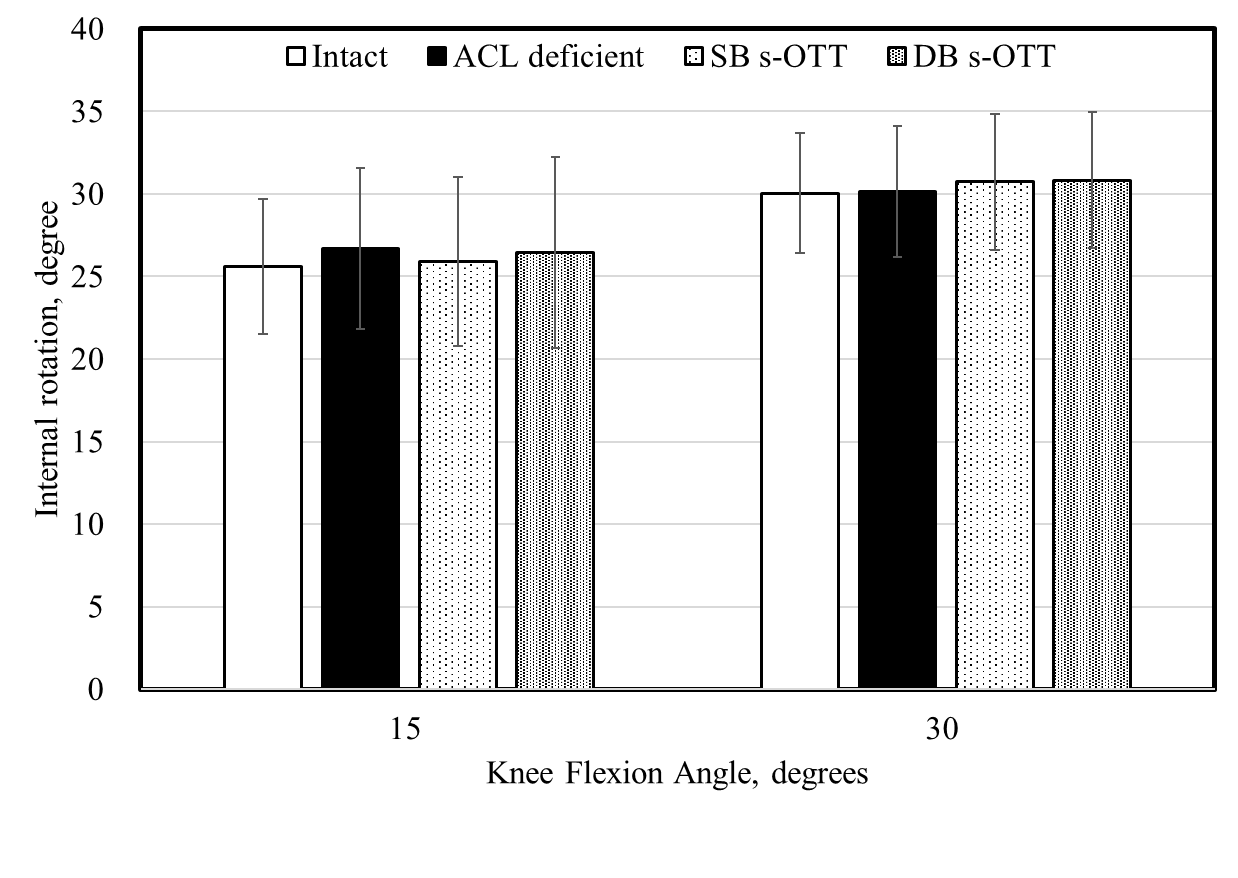


b


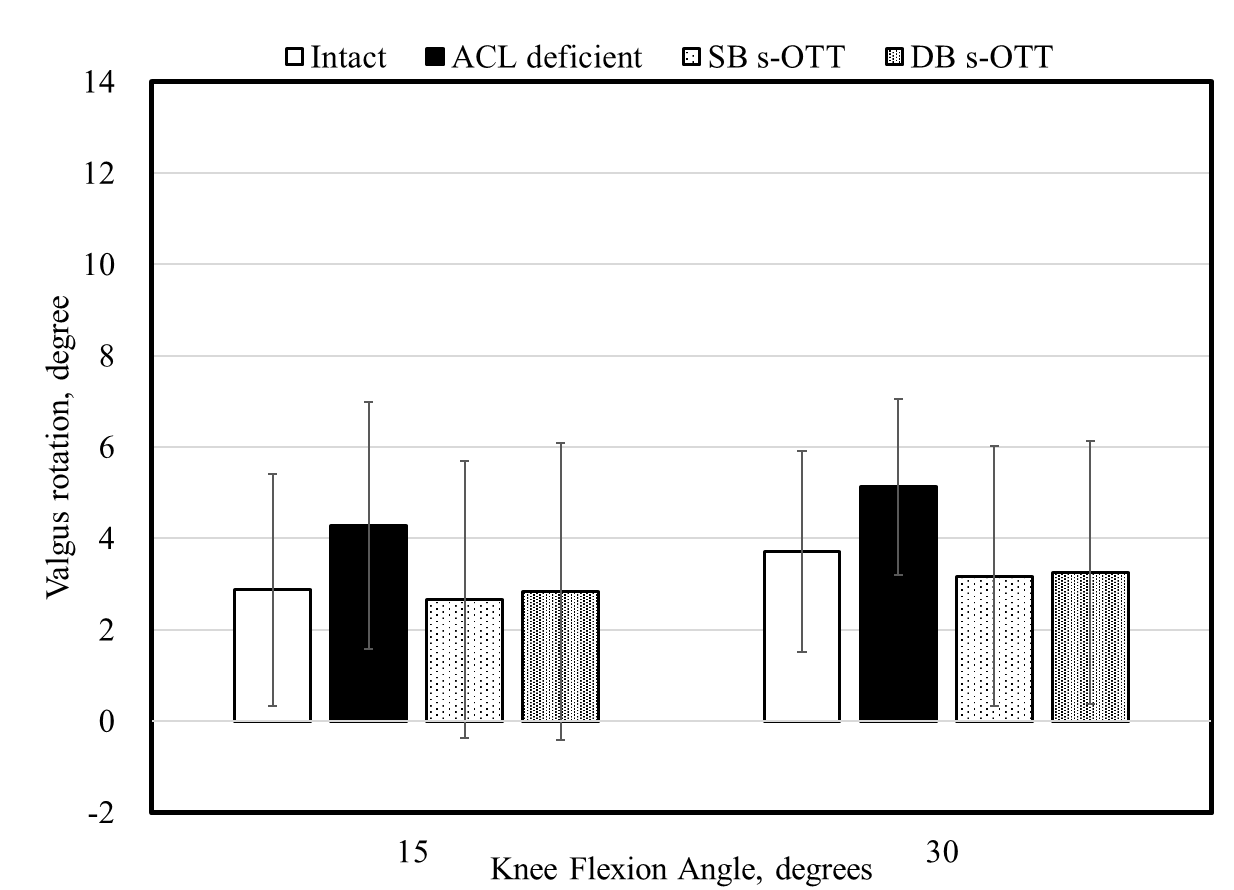


c


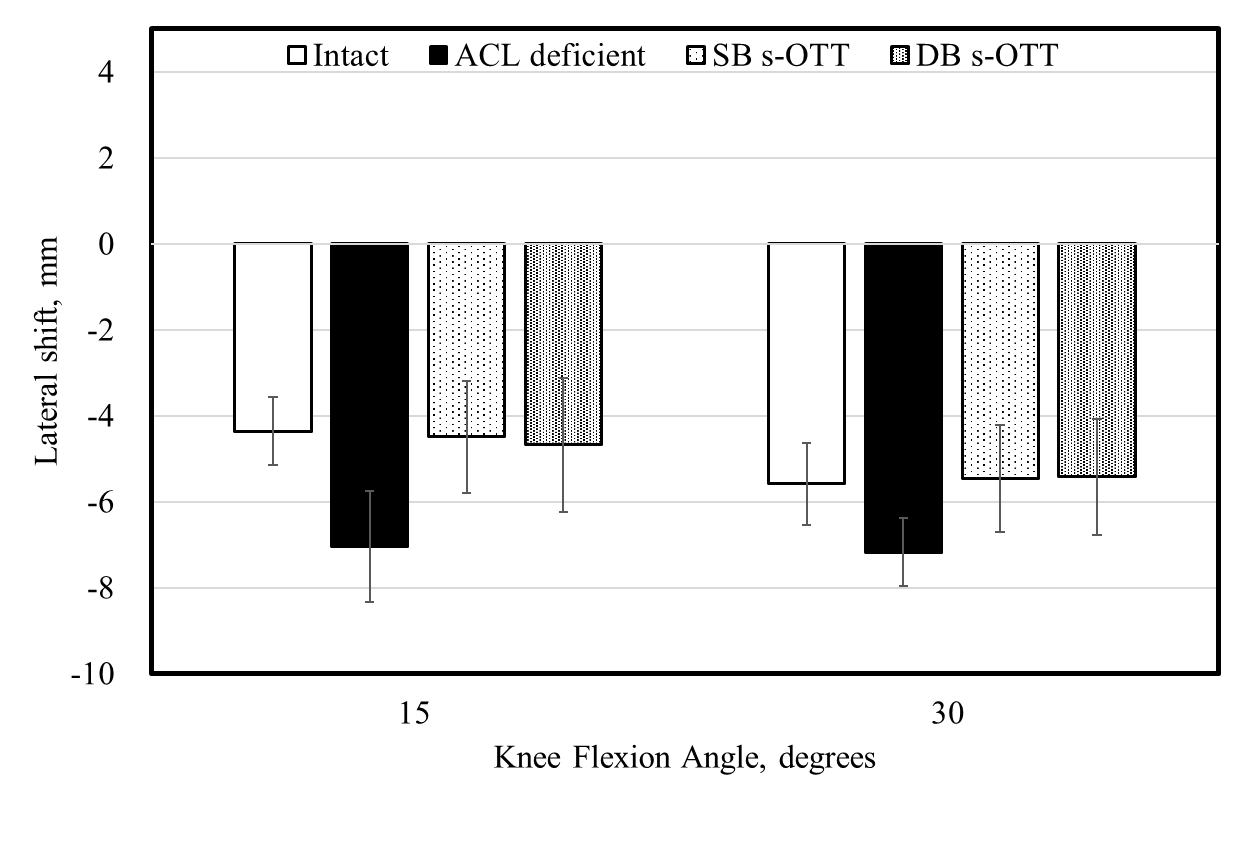


d


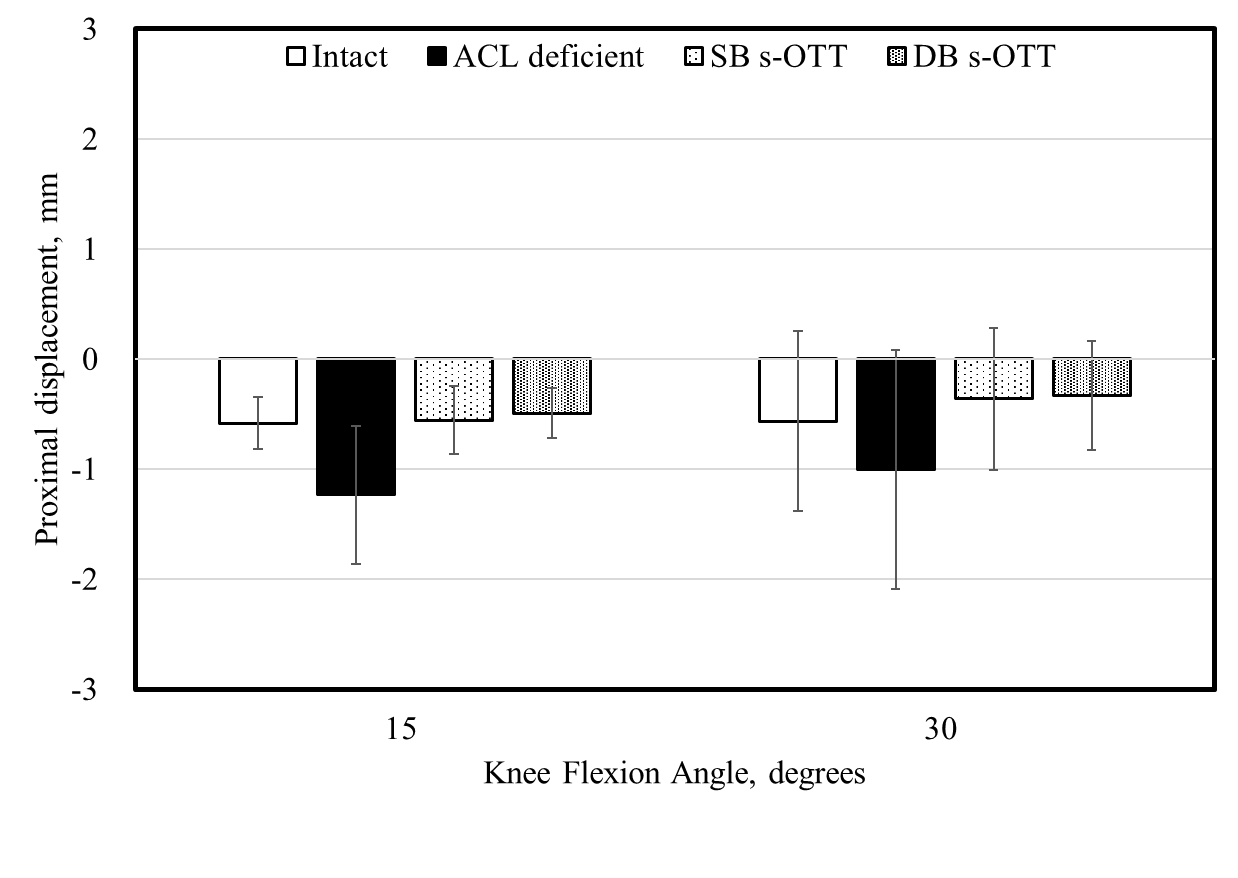

Supplement: Supplementary file 1 — Supplemental Figure 2 Relative position of tibia in each knee status under anterior tibial load. (a) Internal rotation, (b) valgus rotation, (c) lateral shift, and (d) proximal displacement. The error bars indicate 1 SD of the sample mean. ACLR, anterior cruciate ligament; DB s‐OTT, double‐bundle supra‐over‐the‐top; SB s‐OTT, single‐bundle supra‐over‐the‐top. [file JEO2-12-e70466-s002.docx]

**Supplenmental Fig. 1**

a


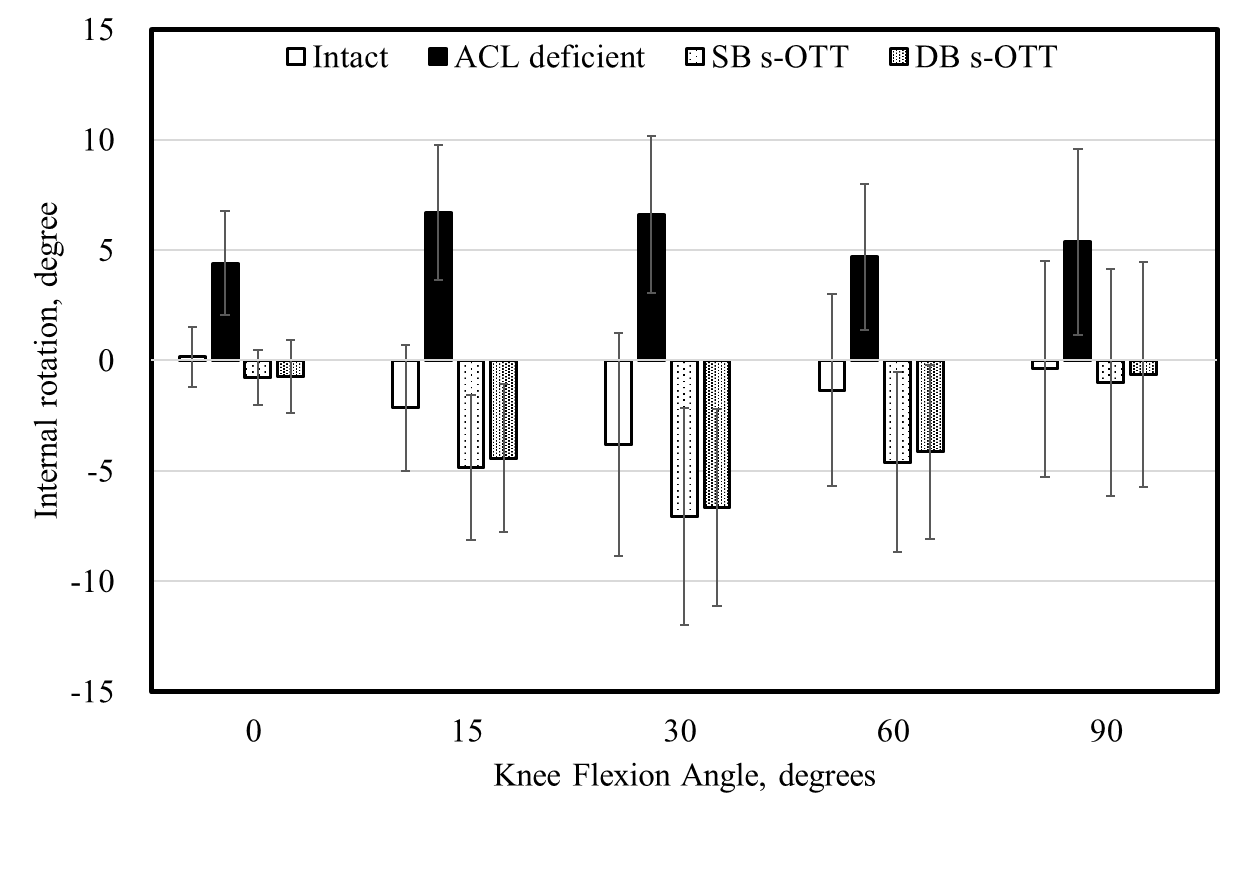


b


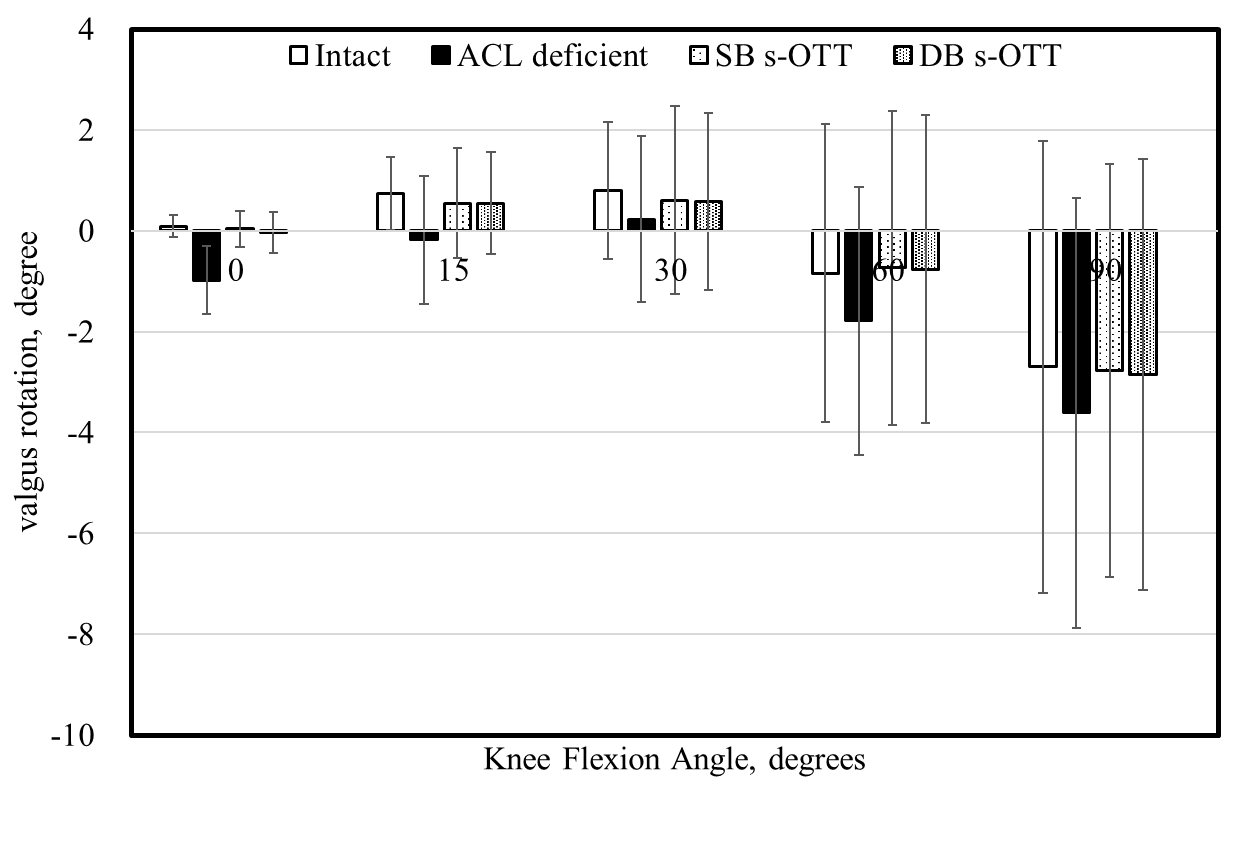


c


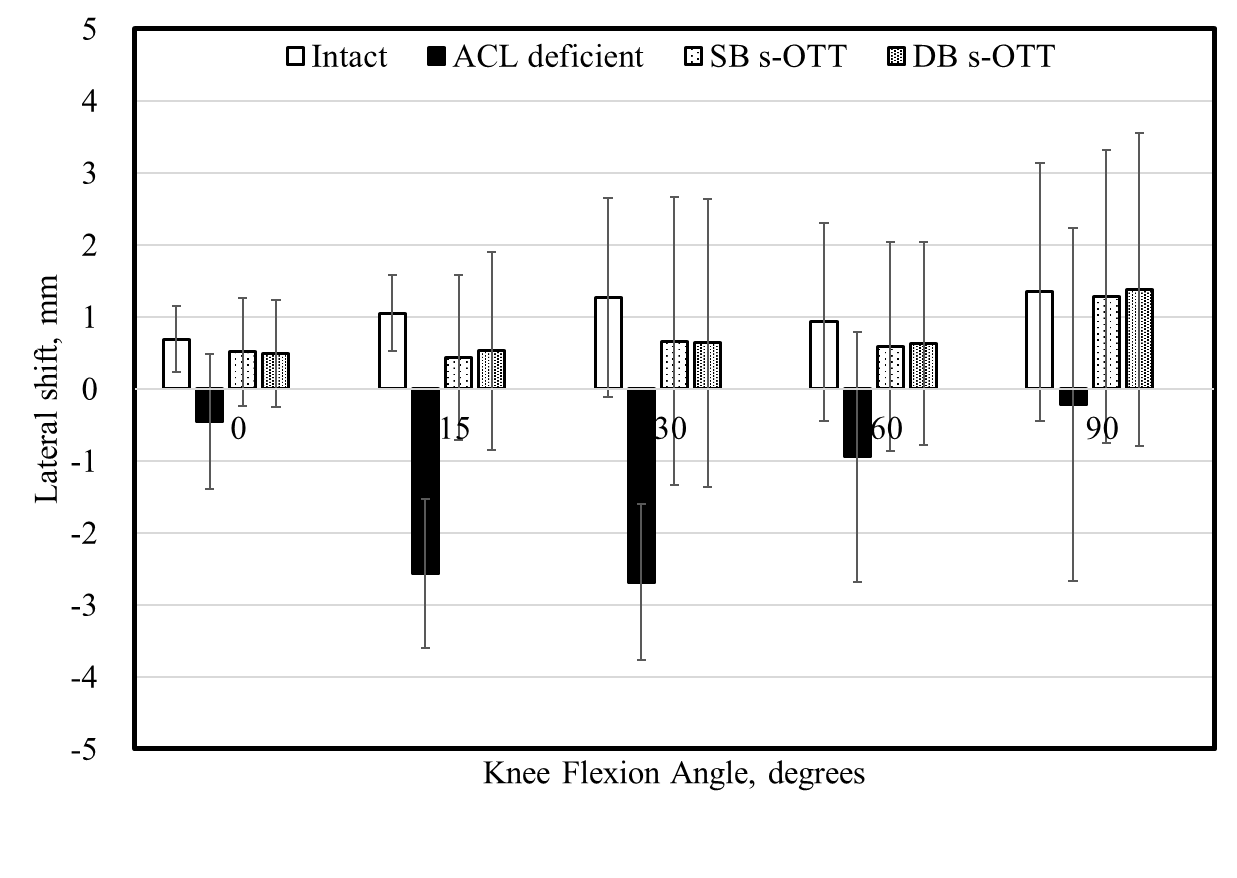


d


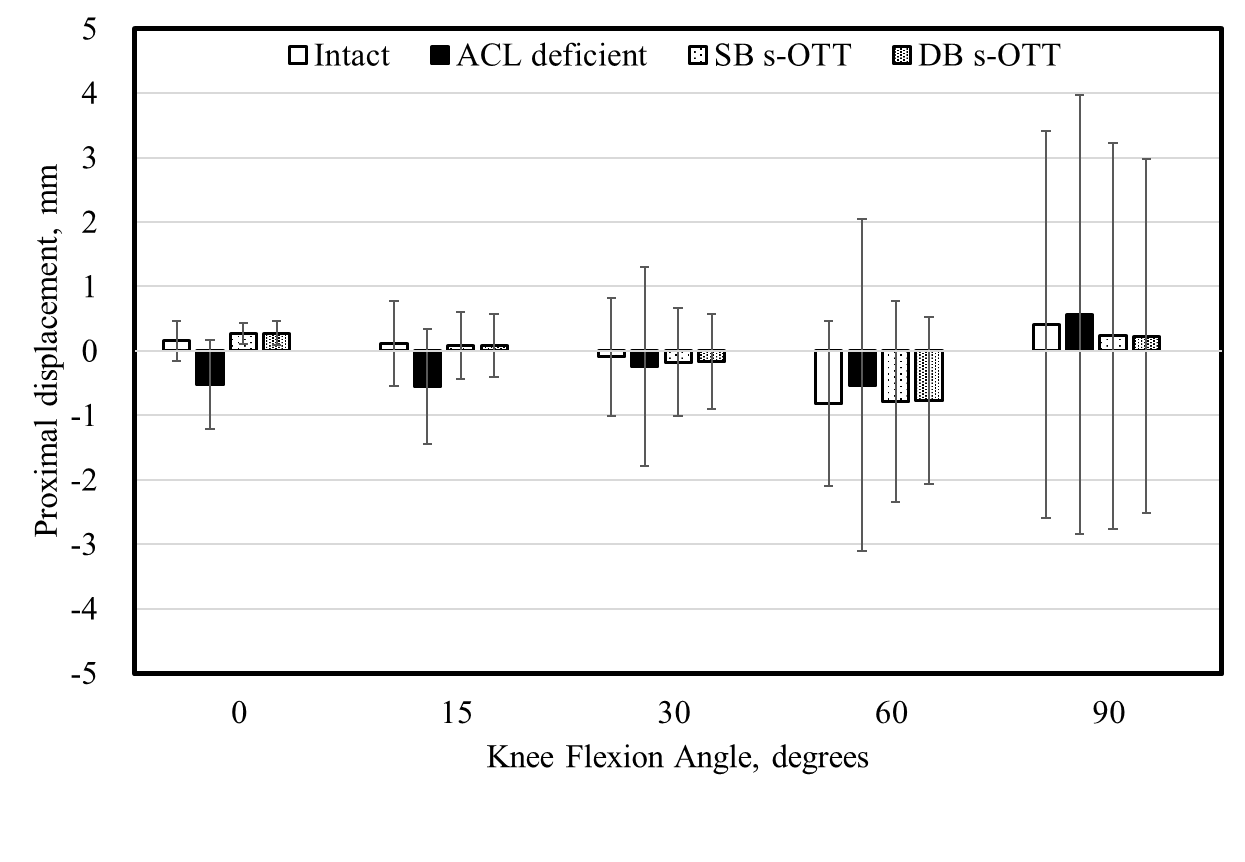

Supplement: Supplementary file 2 — Supplemental Figure 1 Relative position of tibia in each knee status under anterior tibial load. (a) Internal rotation, (b) valgus rotation, (c) lateral shift, and (d) proximal displacement. The error bars indicate 1 SD of the sample mean. ACLR, anterior cruciate ligament; DB s‐OTT, double‐bundle supra‐over‐the‐top; SB s‐OTT, single‐bundle supra‐over‐the‐top. [file JEO2-12-e70466-s001.docx]
